# Reservoir impoundment-triggered seismicity in Brazil: the case of M4.0 Nova Ponte earthquake

**DOI:** 10.1038/s41598-023-48924-6

**Published:** 2023-12-14

**Authors:** Haris Raza, Iman Rahimzadeh Kivi, George Sand França, Victor Vilarrasa

**Affiliations:** 1 Seismological Observatory, Graduate Program in Geology, Institute of Geosciences, University of Brasília, Campus Darcy Ribeiro, Brasília, 70297-400 Brazil; 2 Institute of Astronomy, Geophysics and Atmospheric Sciences, University of São Paulo, São Paulo, 05508-090 Brazil; 3 Global Change Research Group (GCRG), IMEDEA, CSIC-UIB, 07190 Esporles, Spain; 4 Associated Unit: Hydrogeology Group (UPC-CSIC), Barcelona, Spain; 5 Institute of Environmental Assessment and Water Research (IDAEA), Spanish National Research Council (CSIC), Jordi Girona 18-26, 08034 Barcelona, Spain; 6 Present Address: Department of Earth Science and Engineering, Imperial College London, London, SW7 2AZ UK

**Keywords:** Hydrology, Seismology, Natural hazards

## Abstract

Reservoir-triggered seismicity commonly occurs as a result of reservoir impoundment. In particular, the Nova Ponte reservoir triggered a series of earthquakes, including the 1998 M4.0 earthquake, which represents the second-largest earthquake triggered by reservoir impoundment in Brazil. The earthquake occurred after prolonged seismic activity following reservoir impoundment starting in 1993. After more than two decades, the mechanisms governing these earthquakes and their relation with the spatiotemporal evolution of the seismic events are still poorly understood. Here, we explain the causal mechanisms of the two largest earthquakes: an initial response M3.5 in 1995 and the delayed M4.0 in 1998. To this end, we numerically simulate the poromechanical subsurface response to reservoir impoundment using a 3D model that includes three geological layers down to 10 km depth. From the proposed potential nodal planes of the 1995 M3.5 earthquake, we show that the earthquake has most likely occurred on a vertical, E–W-oriented strike-slip fault with a reverse-displacement component. Deviatoric stresses generated by the water column loading on the surface, superimposed by undrained pore pressure enhancement in deep low-permeability layers can explain the fault reactivation. We find that for the 1998 M4.0 earthquake to occur, conductive flow pathways with permeability as high as 6.6·10^−15^ m^2^ should exist to transmit pore pressure to a deep critically oriented fault. Our analysis raises the importance of accounting for coupled poromechanical mechanisms controlling fault stability, hydromechanical properties of different rock layers, and realistic shape of the reservoir to accurately assess the potential for reservoir-triggered seismicity. We conclude that reliable forecasting models require accurate subsurface characterization before reservoir filling to enable managing the associated reservoir-triggered seismicity.

## Introduction

The role of impounding artificial water reservoirs in inducing seismicity deep underground has been studied for several decades^1,2^. Reservoir-triggered seismicity (RTS) has been frequently documented at passive margins in the United States and South America and within stable cratons in Canada and Africa, among which 24 earthquakes had magnitudes > 5, large enough to cause damage^3^. Attempts to understand RTS have come up with two main causal mechanisms^2,4,5^: (1) instantaneous elastic stress transfer by the added mass of water in the reservoir; (2) time-dependent diffusion of pore pressure into the subsurface. The former mechanism can alter the shear stress resolved on pre-existing deep faults and change the pore pressure by compaction of the pore space, i.e., undrained loading. The pore pressure build-up by the latter mechanism gradually decreases the effective normal stress acting on faults and, thus, their friction resistance against slip. The changes in stresses and pore pressure may cause shear rupture on critically stressed faults and induce earthquakes^1,6^.

Elastic stress transfer and pore pressure diffusion mechanisms were successfully invoked to describe several earthquakes connected to reservoir impoundment, including the 1967 M6.3 Koyna earthquake in India^7–9^, the 1975 M5.7 Oroville earthquake in the USA^10^, the 2004 M4.7 Itoiz earthquake in Spain^11^, and the 1994 M3.0 Açu reservoir in Brazil^12^, just to name a few high-profile cases. Insights gained from these studies show that pore pressure and stress changes in the order of a few tenths of MPa are enough to reactivate deep faults^1,13^. Furthermore, the occurrence and distribution of seismicity in time and space were found to follow complex patterns: earthquakes may occur at the early stages of reservoir impoundment or be delayed by several years or decades^2^. The latter case commonly corresponds to deeper and larger seismic events^4^. These complexities in the spatiotemporal distribution of the seismic events depend primarily on the water level in the reservoir, reservoir filling rate, water level fluctuations, in situ stress state and hydrogeological characteristics of the underground formations, among others^13–15^. Therefore, understanding the interactions between these factors would be the key to improved prediction and mitigation of the RTS hazard.

**Figure 1:**
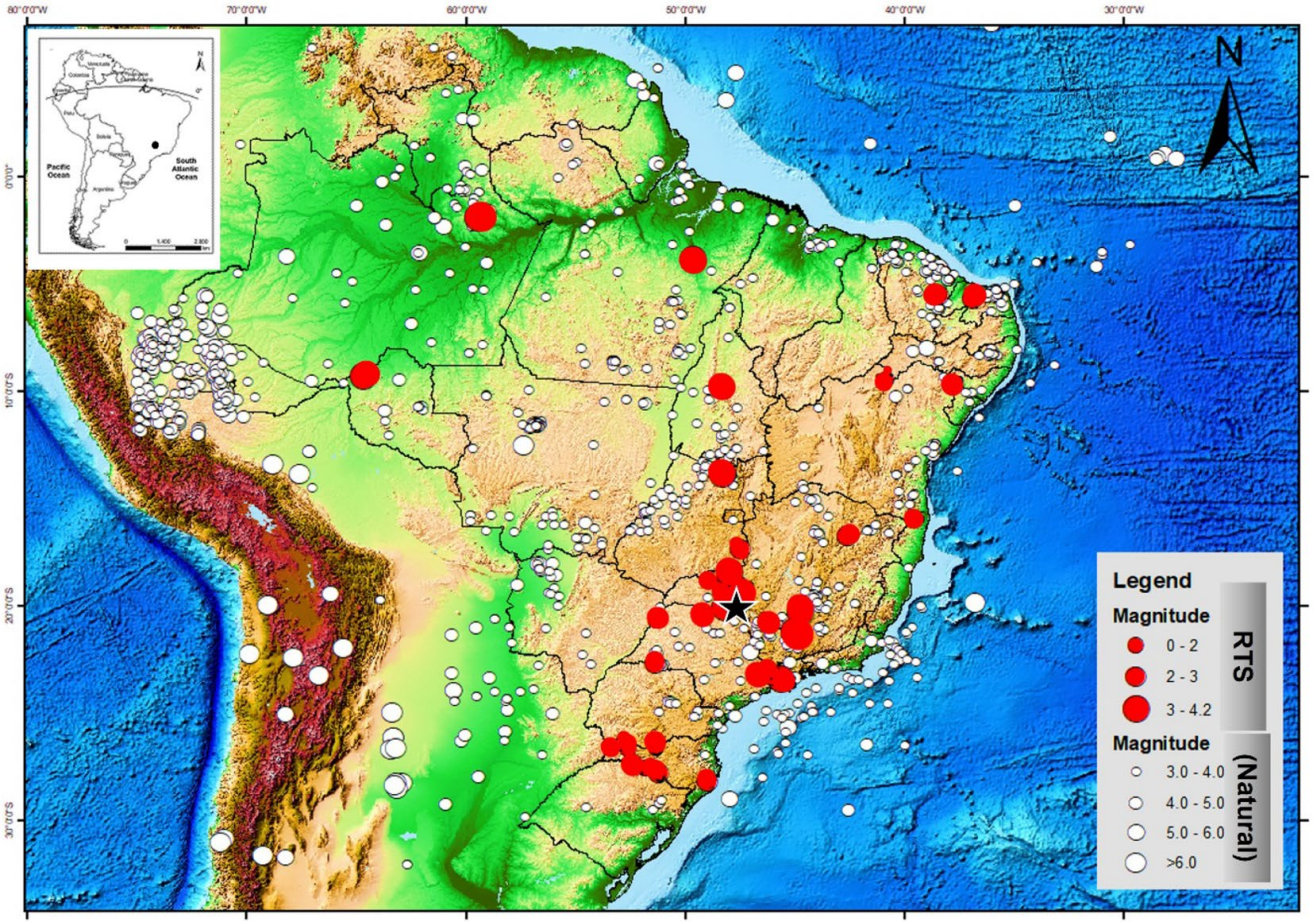
Brazil map showing RTS (Reservoir-triggered seismicity) in red circles and natural earthquakes in white circles, with size proportional to magnitude and the black star showing the main event of Nova Ponte. The data obtained from the bulletin of the IAG-USP and SISBRA-UnB (modified after Sayão et al.^16^).

**Figure 2:**
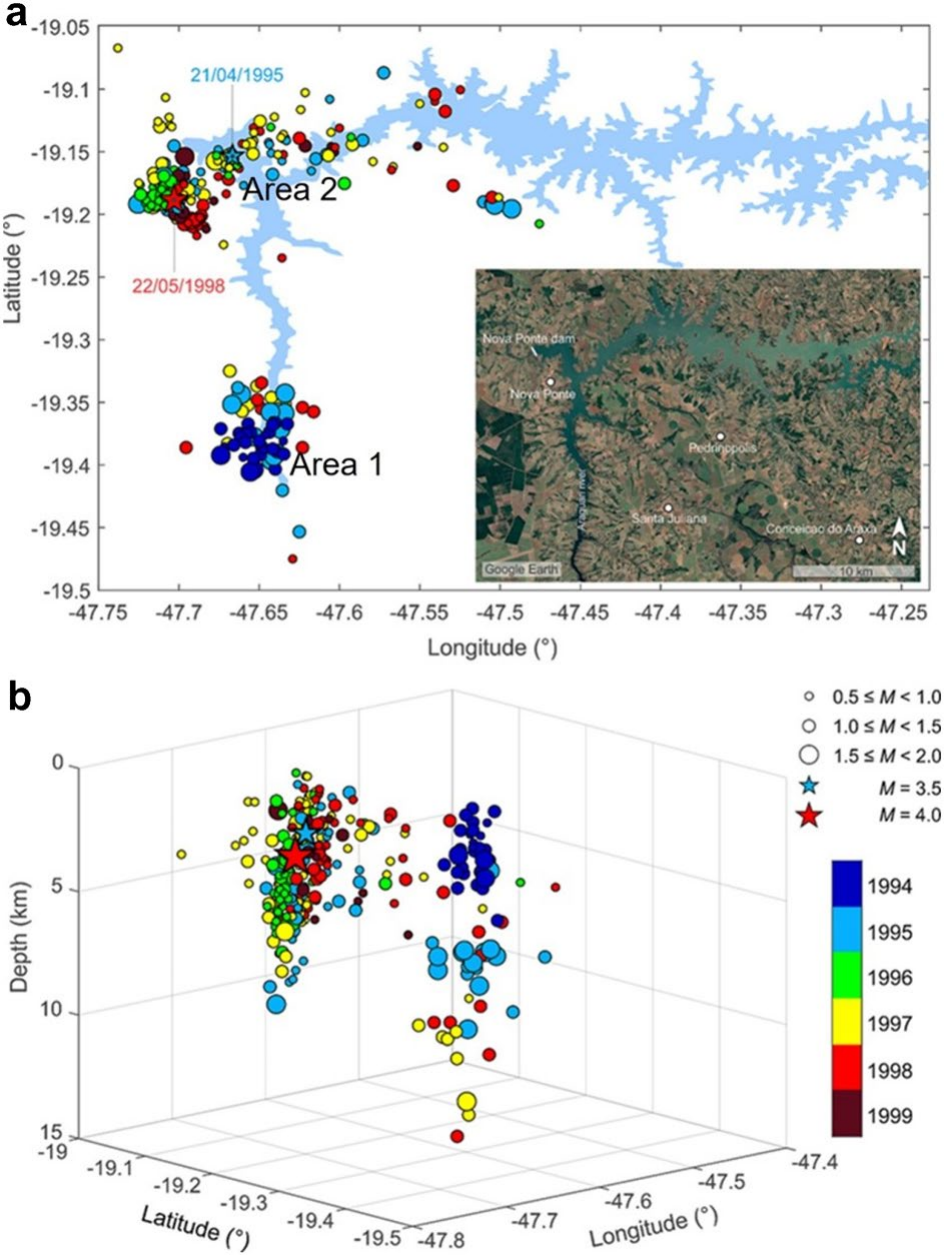
(**a**) Epicentral location of the Nova Ponte reservoir and seismic events M ≥ 0.5 of Areas 1 and 2 (**b**) and their distribution with depth epicentral error ranging from 0.3 to 1.5 km color-coded by year from 1994 to 1999. The inset in (**a**) shows the reservoir location acquired from Google Earth.

The Nova Ponte reservoir in South-eastern Brazil was the location of a M4.0 reservoir-triggered earthquake, the second largest RTS in Brazil since the first recording in 1972^16,17^ (Fig. 1). The reservoir is located in the state of Minas Gerais. The dam of the Nova Ponte hydropower plant lies in the close vicinity of Nova Ponte city (Fig. 2a). The M4.0 earthquake at Nova Ponte occurred in 1998 after prolonged seismic activity following reservoir impoundment in 1993. The relatively large magnitude of the earthquake and the long-time lag after reservoir impoundment has raised questions about the triggering mechanisms of this RTS. Reliable knowledge of these mechanisms, although of paramount importance to assure the safety of infrastructure at the Nova Ponte reservoir and the local population, has not been gained yet.

Various models have been proposed to delineate the main causes of initial and delayed RTS at various sites^1,2,10,18–20^. Table 1^1,2,10,19–23^ outlines the important features of these models. Three types of poroelastic coupling are considered in the literature: (1) coupled, in which poroelastic stresses influence pore pressure and vice versa; (2) uncoupled, in which poroelastic stresses and pore pressure do not affect each other; and (3) decoupled, in which poroelastic stresses change pore pressure, but not conversely^1^. Most of the recent models simulate pore pressure diffusion in a homogeneous 2-D medium with constant-permeability faults represented as 1-D features embedded in a 2-D half-space. Subsequently, 3D models have been used, like the one of Monticello reservoir, South Carolina^20^, which illustrated that pore-pressure diffusion is the governing mechanism for RTS even during reservoir filling at this site. Yet, the vast majority of models simplify the subsurface, homogenising the properties of the different rock layers.

**Table 1:** Reservoir-triggered seismicity models and their characteristics.

Model	Investigated response	Media properties and geometry
Bell and Nur^10^	Decoupled	Homogeneous rock matrix, homogeneous fault 2D
Roeloffs^1^	Coupled and decoupled	Homogeneous rock matrix 2D
Talwani^2^	Coupled and decoupled	Homogeneous rock matrix 2D
Gahalaut and Chander^21^	Decoupled	Homogeneous rock matrix 3D
Kalpna^19^	Decoupled	Homogeneous rock matrix 3D
Chen and Talwani^20^	Decoupled	Homogeneous rock matrix 2D
Do-Nascimento et al.^22^	Decoupled	Homogeneous rock matrix, homogeneous fault 3D
Gavrilenko et al.^23^	Coupled	Homogeneous rock matrix, homogeneous fault 3D

The aim of this study is to investigate the causal mechanisms of the main events at Nova Ponte. We first present the geological setting where the reservoir is located. Then, we explain the characteristics of the RTS at Nova Ponte, including the temporal evolution of the seismicity in the period 1994–1999 and the location of the main events based on the local velocity model. To assess the contribution of elastic stress transfer and pore pressure diffusion at Nova Ponte, we build a coupled 3D reservoir model including three rock layers and simulate the poroelastic response of the subsurface to the reservoir impoundment. Finally, we present our main findings and our interpretation of the RTS at Nova Ponte.

## Geological setting and seismicity

### Geological setting

The Nova Ponte reservoir is located at the Paraná Basin, a Phanerozoic volcano-sedimentary basin, at the boundary with another geotectonic province named the Neoproterozoic Brasilia Fold Belt. Several rock types are present in the study area, including granite-gneissic complex (Archaean-Paleoproterozoic), the Canastra and Paranoá groups (Meso/Neoproterozoic) and the Araxá, Ibiá and Bambuí groups (Neoproterozoic).

The lithology (rock layers) are, from top to bottom, meta basalt (Serra Geral Formation), Araxá Group rock and the crystalline basement^24^. The Serra Geral Formation is predominantly constituted by basaltic flows that are superimposed on the Botucatu Sandstone formation. The basalt has its origin in intracontinental volcanism from 100 to 140 Ma ago. The volcanic rock types of the Serra Geral Formation are composed of hard and massive fine-grained basalts and massive amygdales structures^25^. The Araxá Group of the Neoproterozoic age basically consists of schist, shale, micaceous quartzite. The presence of volcanic rocks indicates the volcano-sedimentary character of the Araxá sequence^26^. Some intrusions of amphibolite gabbroid bodies can also be observed. The main structural pattern of this unit is the low-angle foliation in the N-W direction, which is associated with tectonic transport towards the San Francisco Craton^25,27^. The granite-gneiss complex is mainly composed of banded, micaceous, and quartz-feldspathic, massive and gneissified granite^25^.

### Background on the triggered seismicity at Nova Ponte

The Nova Ponte dam has a total height of 142 m, keeping a reservoir area of 443 km^2^ with a reservoir volume of 12.8 km^3^. The dam was constructed on the Araguari rivers, filling the reservoir to a maximum height of 132 m. The filling of the reservoir began in October 1993. The very first triggered seismic events were detected when the reservoir filled to approximately 18% of its total capacity. A strong association between earthquake activity and loading/unloading of the reservoir has been observed. The events started in January 1994, being located mainly in the southern part of the reservoir (Fig. 2) and were felt by the local population^28^.

**Figure 3:**
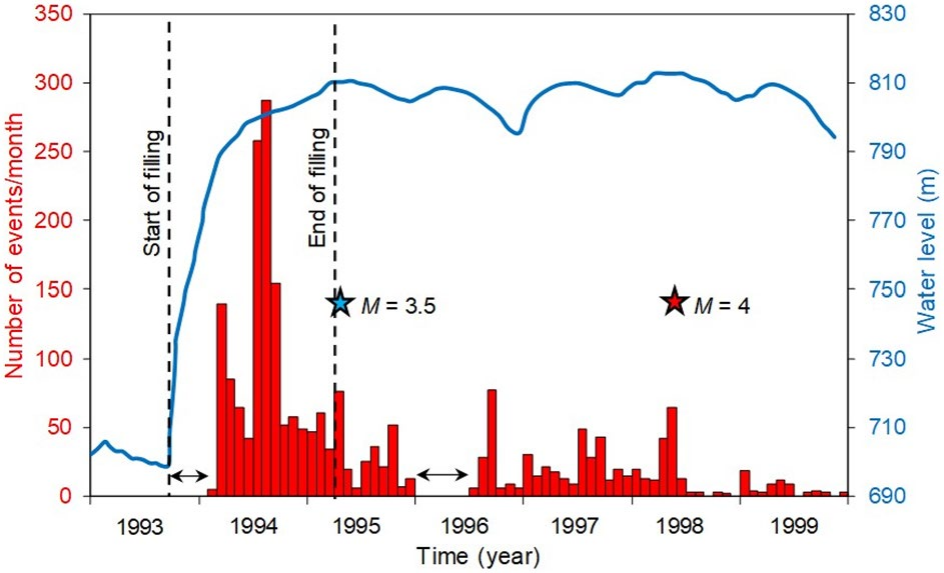
Temporal evolution of RTS at Nova Ponte. Number of monthly events (histogram) and monthly average water elevation above the mean sea level (blue line) are illustrated. Stars point to the two largest events (initial and delayed). Arrows indicate the two periods without data when the stations were down^37^ (modified after Assumpção et al.^28^^,^^37^).

The largest event of the initial phase of seismicity had a magnitude M3.5 and was nucleated at a depth of 2.6 km. This event occurred approximately 3 to 4 km east of the dam about 1.5 years after starting reservoir impoundment (on 21.04.1995) (Fig. 2), when the water reached the level of 110.6 m (Fig. 3). Seismic activity continued near the dam (Fig. 2) and the main event of M4.0 occurred further away from the reservoir margin due to a delayed response 4.5 years after impoundment (on 22.05.1998) (Table 2). The depth of this event was approximately 3 km (Table 2).

**Table 2:** Characteristics of triggered seismicity at Nova Ponte^16^.

Dam height (m)	Volume (km^3^)	Water depth (m)	Reservoir area (km^2^)	Seismicity type	Date	Location	Magnitude (mR)	Io (MMI)	∆T (year)	Depth of earthquake (km)
142	12.8	132	443	Initial	21.04.1995	Margin	3.5	IV–V	1.5	~ 2.6
				Delayed	22.05.1998	Outside	4.0	VI	4.5	~ 3

∆T: time interval (years) since the beginning of filling/impoundment of the reservoir; MMI: modified Mercalli scale.

The events location for Area 1 are grouped into a region of approximately 5 km in radius. The epicenters show poor alignment in the NNW direction in 1994 and there was a migration from a restricted area near the reservoir to the vicinity of the margin in 1998. A striking feature is the increase in the depth and lateral dispersion of the hypocenters over time, showing both vertical and lateral diffusion. The epicenters of these events are distributed between 0 and 15 km in depth, presenting a progressive increase in depth. Major events of Area 1 (M > 1.5–2.0) were deeper than 5 km in 1994, mostly between 5–10 km in 1995, and between 7–14 km in 1997 (Fig. 2).

The events in Area 2 are grouped in a cluster delimited by an area of approximately 4 km wide and 7 km long. This cluster contains the two largest events, the initial M3.5 earthquake in 1995 and the delayed M4.0 event in 1998, as well as the earthquakes associated with them, i.e., fore- and after-shocks. The uncertainty of earthquakes was generally less than 0.1 of the root mean square residual, both in horizontal and depth errors. In this work, we did not consider this factor as an influencing element in the modeling process.

The velocity model to locate seismic events in the Nova Ponte area was based on a deep seismic refraction survey^29^ in combination with local geological interpretations and studies of the crustal structure in south-eastern Brazil^28^. The velocity model consists of a superficial 0.3-km-thick layer with a P wave velocity (Vp) of 5.0 km/s, representing the basalts of the Serra Geral Formation. The second layer has a thickness of 5.7 km and a Vp of 5.7 km/s and is related to the Araxá Group schist. The basement is located below these two layers, with a Vp of 6.1 km/s. A compressional-to-shear velocity ratio (Vp/Vs) of 1.7 has been considered for these layers^28^. Accordingly, two different sets of fault plane solutions for the April 21, 1995, M3.5 earthquake have been proposed (Table 3): one points to reverse faulting with a strike-slip component^30^, and the other mainly includes a strike-slip fault^31^. However, the accuracy of these focal mechanisms remains debated because of the low quality of the recorded seismic data by analog seismograms and uncertainties associated with the velocity model. Due to the same reasons, focal mechanisms of the May 22, 1998, M4.0 earthquake have not yet been resolved.

**Table 3:** Proposed focal mechanisms of the 1995 M3.5 earthquake at Nova Ponte.

Strike angle	Dip angle	Rake angle	References
85	38	20	Assumpção et al.^30^
190	46	168	Marza et al.^31^
272	89	42	Marza et al.^31^

## Poroelastic modeling of fault stability

**Figure 4:**
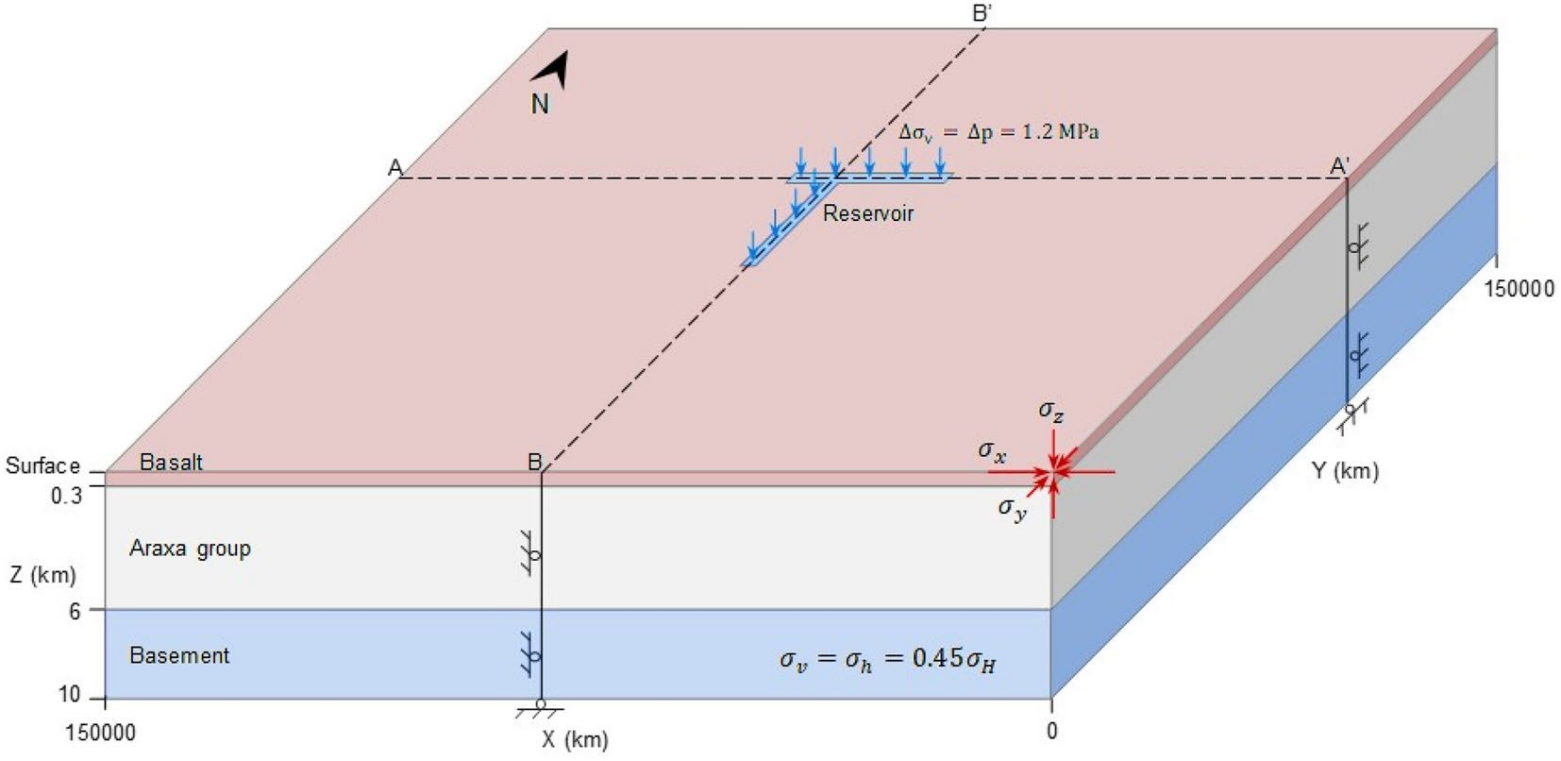
Model geometry and boundary conditions used in this study to understand the triggering mechanisms of the Nova Ponte RTS.

We perform coupled hydromechanical numerical simulations of the Nova Ponte reservoir impoundment to understand whether and how the resulting pore pressure and stress perturbations caused earthquakes at depths. We consider a conceptual 3D model of the upper 10 km of the crust, including the basalt layer, extending from the surface to a depth of 300 m, the 5.7-km thick Araxá group, and a 4-km thick portion of the crystalline basement at the bottom^29^ (Fig. 4). Neglecting topography, the reservoir cross-section area is reproduced as a T-shape surface on the top of the basalt layer. The water in the reservoir is not modelled, but a hydrostatic pressure and an overburden stress of equivalent weight are applied on the top of the reservoir cross-section. The model extends more than 50 km on each side of the reservoir in the x and y directions to exclude potential boundary effects.

We assume that all geological media are homogeneous and isotropic and deform elastically. Given the lack of direct measurements of the hydraulic and mechanical properties of the modelled layers, we assign typical values of the considered rock types (Table 4)^32,33^. Initial conditions include hydrostatic pressure distribution with a gradient of 0.01 MPa/m. The resolved focal mechanism of the M3.5 earthquake yields a strike-slip/reverse faulting stress regime, with a maximum horizontal stress direction ranging from 42° to 66°, which are consistent with the regional tectonics^34^. Accordingly, the initial vertical stress, *σ*_*v*_, is calculated with the average bulk density of the rock layers and is taken equal to the minimum horizontal stress, *σ*_*h*_, with *σ*_*v*_ = 0.45 *σ*_*H*_, where *σ*_*H*_ is the maximum horizontal stress. We perform a steady-state calculation to derive distributions of the pore pressure and stresses at equilibrium.

**Table 4:** Static mechanical and hydraulic properties for common rock types used in the numerical simulations^32,33^.

Rock types	Basalt	Schist ⊥	Granite
Rock density, *ρ* × 103 (kg/m^3^)	2.8	2.81	2.7
Young’s modulus, *E* (GPa)	60	20	70
Poisson’s ratio, *ν* (−)	0.25	0.3	0.25
Intrinsic permeability, *k* (m^2^)	10^–15^	10^–17^	10^–19^
Porosity*, ϕ* (−)	0.05	0.1	0.01

⊥ refers to the parameters measured perpendicular to the bedding planes.

We simulate reservoir impoundment by increasing the pore pressure and vertical stress on top of the reservoir section over 1.5 years from atmospheric conditions to 1.3 MPa, coinciding with the final water column height of about 120 m. We extend the simulation to 10 years by maintaining the stress and pressure conditions in the reservoir to address the post-impoundment period. The pressure and stress conditions in the area surrounding the reservoir are kept atmospheric throughout the simulation. We impose no-flow boundary conditions on the bottom and lateral boundaries. Mechanical boundary conditions comprise no displacement perpendicular to these boundaries. We numerically solve the described hydromechanical problem using the fully-coupled finite element code CODE_BRIGHT^35^. The mesh consists of 55 × 46 × 55 (139,150 in total) hexahedral elements, which are both vertically and horizontally refined toward the reservoir. The spatiotemporal distributions of the pore pressure and stresses are used to estimate the fault stability evolution on the orientation of the M3.5 earthquake by adopting the Coulomb Failure Stress, CFS = τ ˗ μσ_n_', where τ and σ_n_' are the shear and effective normal stresses acting on the fault plane, respectively. The fault is assumed to be cohesionless and have a frictional coefficient μ of 0.6^36^ Fault slip is promoted if the CFS increases (i.e., ΔCFS > 0) and hindered if CFS decreases (i.e., ΔCFS < 0).

**Figure 5:**
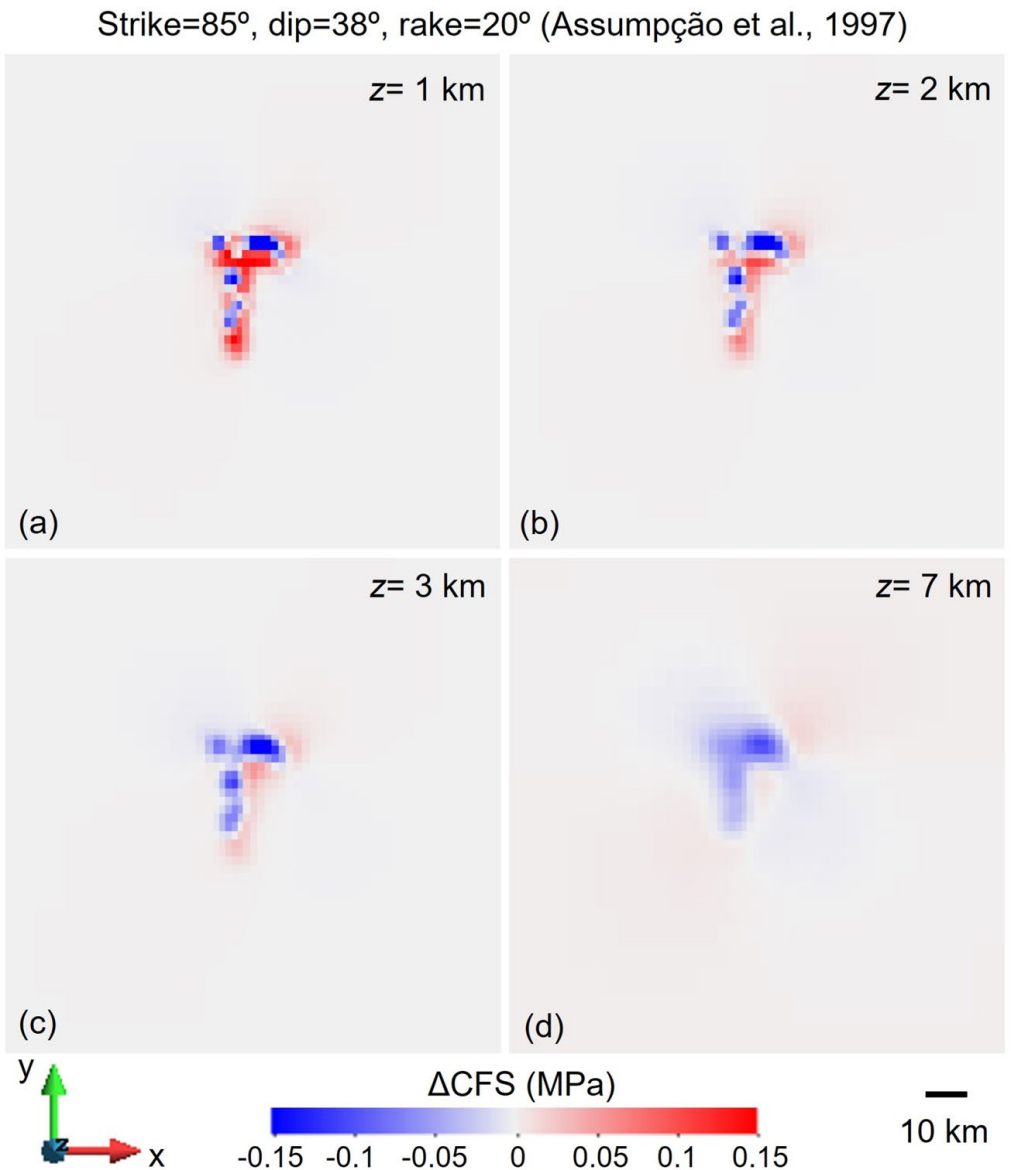
Changes in Coulomb Failure Stress (ΔCFS) for the nodal plane with strike 85º, dip 38º and rake 20° proposed^30^ at the time of the 1995 M3.5 earthquake at (**a**) 1 km, (**b**) 2 km, (**c**) 3 km and (**d**) 7 km depth horizontal cross sections, for the case of schist permeability equal to 8·10^–15^ m^2^ and assuming an orientation of the maximum horizontal stress with respect to the North of 42°. The ΔCFS has negative values at the location of the earthquake, i.e., south of the dam at 2.6 km depth.

**Figure 6:**
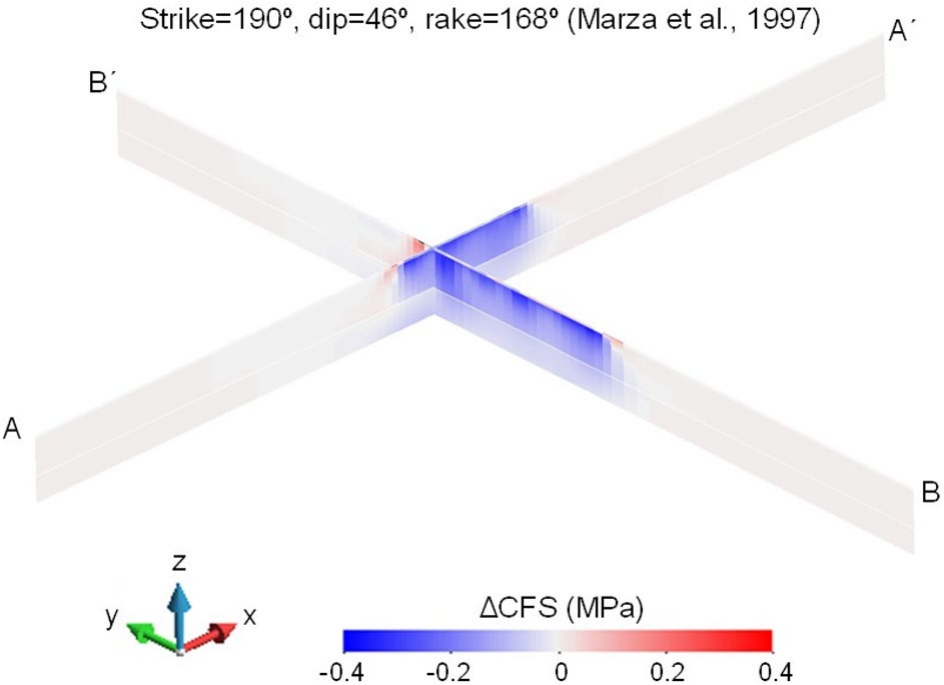
Changes in Coulomb Failure Stress (ΔCFS) for the nodal plane with strike 190°, dip 46° and rake 168º proposed by Marza et al.^31^ at the time of the 1995 M3.5 earthquake below the reservoir along the N–S and E–W directions (corresponding to sections BB´ and AA´ in Fig. 4, respectively), assuming an orientation of the maximum horizontal stress with respect to the North of 42°.

**Figure 7:**
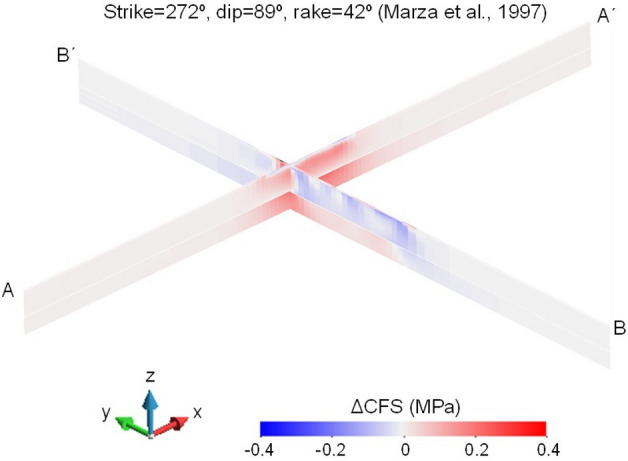
Changes in Coulomb Failure Stress (ΔCFS) for the nodal plane with strike 272°, dip 89° and rake 42° proposed by Marza et al.^31^ at the time of the 1995 M3.5 earthquake below the reservoir along the N–S and E–W directions (corresponding to sections BB´ and AA´ in Fig. 4, respectively), assuming an orientation of the maximum horizontal stress with respect to the North of 62°. The ΔCFS has positive values at the location of the earthquake, i.e., south of the dam at 2.6 km deep.

**Figure 8:**
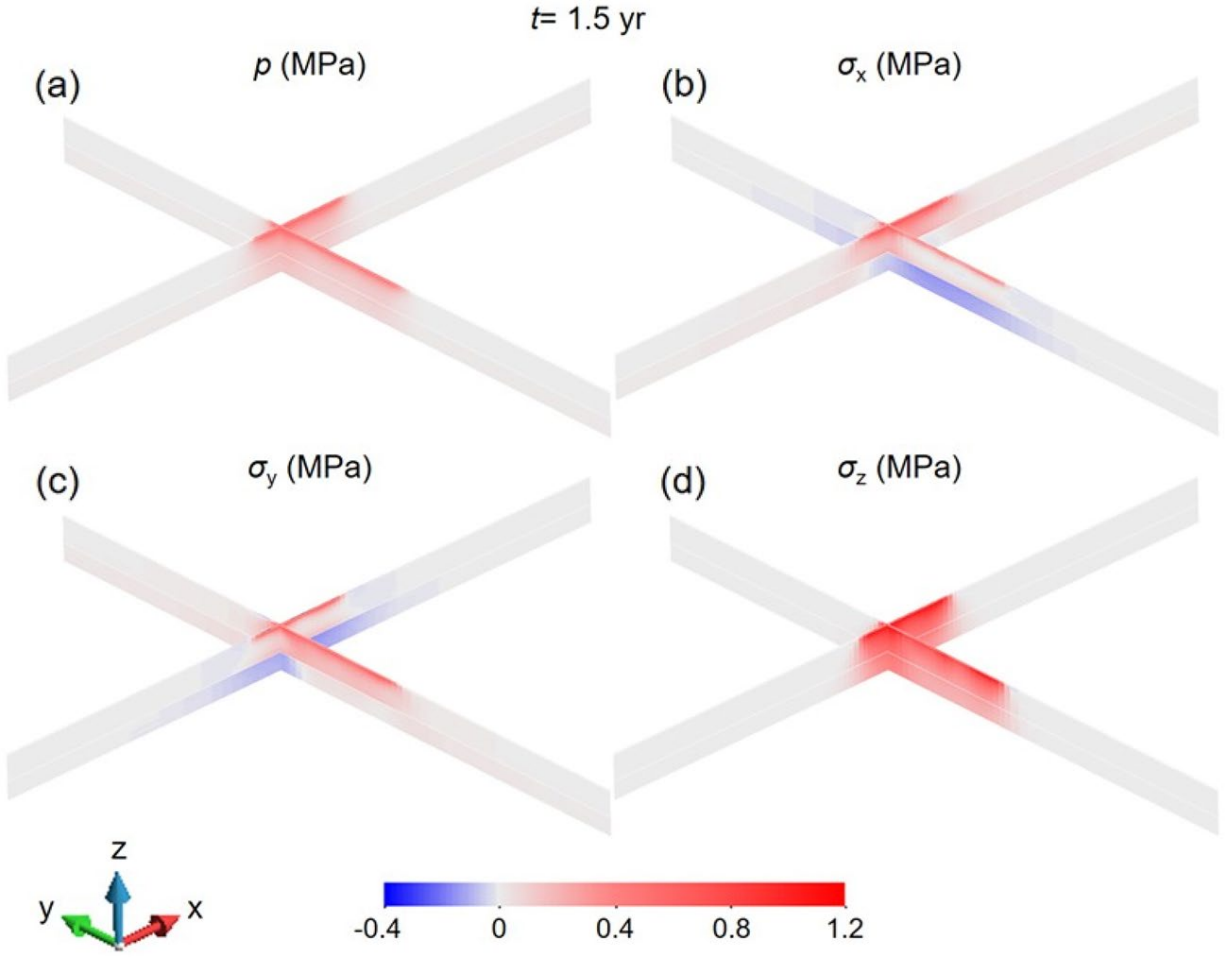
Changes in (**a**) pore pressure, (**b**) horizontal stress in the x direction, (**c**) horizontal stress in the y direction and (**d**) vertical stress at the time of the 1995 M3.5 earthquake below the reservoir along the N-S (y coordinate corresponding to section BB´ in Fig. 4) and E–W directions (x coordinate, corresponding to section AA´ in Fig. 4).

To generate the figures, we have used Matlab version 2023b to plot the earthquake location (Fig. 2), GiD v14 to create the contour plots (Figs. 5, 6, 7, 8 and 10) and Matlab version 2023b and CorelDRAW X7 to plot the correlation between contour plots and seismicity distribution (Fig. 11). Figure 1 has been modified after Sayão et al.^16^ by esri ArcGIS (ide.unb.br/como-usar/) and Fig. 3 has been modified after Assumpção et al.^28^^,^^37^.

## Results

Two focal mechanisms obtained from the initial triggered seismicity occurred in 1995 are available in the literature (Table 3)^30,31^. The nodal plane proposed by Assumpção et al.^30^ corresponds to a reverse fault with strike 85°, dip 38° and rake 20°. The orientation of the maximum horizontal stress (within the range of proposed values in the literature, i.e., azimuth ranging from 42° to 62°) should be 42° to make this plane as much critically oriented for shear slip as possible. At the time of the 1995 M3.5 earthquake, i.e., 1.5 years after the start of the reservoir filling, pore pressure diffusion had not reached the depth of the hypocentre (2.6 km). Therefore, the pore pressure and stress changes occurring at depth are due to the undrained response of the subsurface to the loading of the retained water by the dam. The changes in Coulomb Failure Stress (ΔCFS) are negative at the depth of the hypocentre and thus, the focal mechanism proposed by Assumpção et al.^30^ could not delineate the RTS. We have tested whether an enhanced permeability of the schist of 8·10^–15^ m^2^, sufficiently high to allow the pore pressure propagation front to reach the depth of the earthquake, could explain the seismicity due to the pore pressure build-up at depth. Similarly, to the case of low-permeability schist (10^–17^ m^2^), the ΔCFS presents negative values at the location of the 1995 M3.5 earthquake (Fig. 5).

The other focal mechanism of the 1995 M3.5 earthquake^31^ gives as possible nodal planes a reverse-faulting plane with right-lateral displacement and a strike-slip plane with reverse-left-lateral displacement. To make the nodal planes the most favourably oriented planes for slip within the range of orientations of the maximum horizontal stress, the azimuth of the maximum horizontal stress should be 42° for the reverse-faulting plane with right-lateral displacement and 62° for the strike-slip plane with reverse-left-lateral displacement. By assuming a schist permeability of 10^–17^ m^2^, the pore pressure diffusion does not reach the depth of the hypocentre and thus, the subsurface response to the 1995 M3.5 earthquake would have been undrained. Similarly to the reverse-faulting plane obtained by Assumpção et al.^30^, the ΔCFS of the reverse-faulting plane with right-lateral displacement presents negative values below the reservoir (Fig. 6). In contrast, the strike-slip plane with reverse left-lateral displacement displays positive values of ΔCFS below the reservoir along the E–W direction (Fig. 7). Thus, the 1995 M3.5 earthquake most likely occurred in a vertical fault with a strike in the E–W direction.

**Figure 9:**
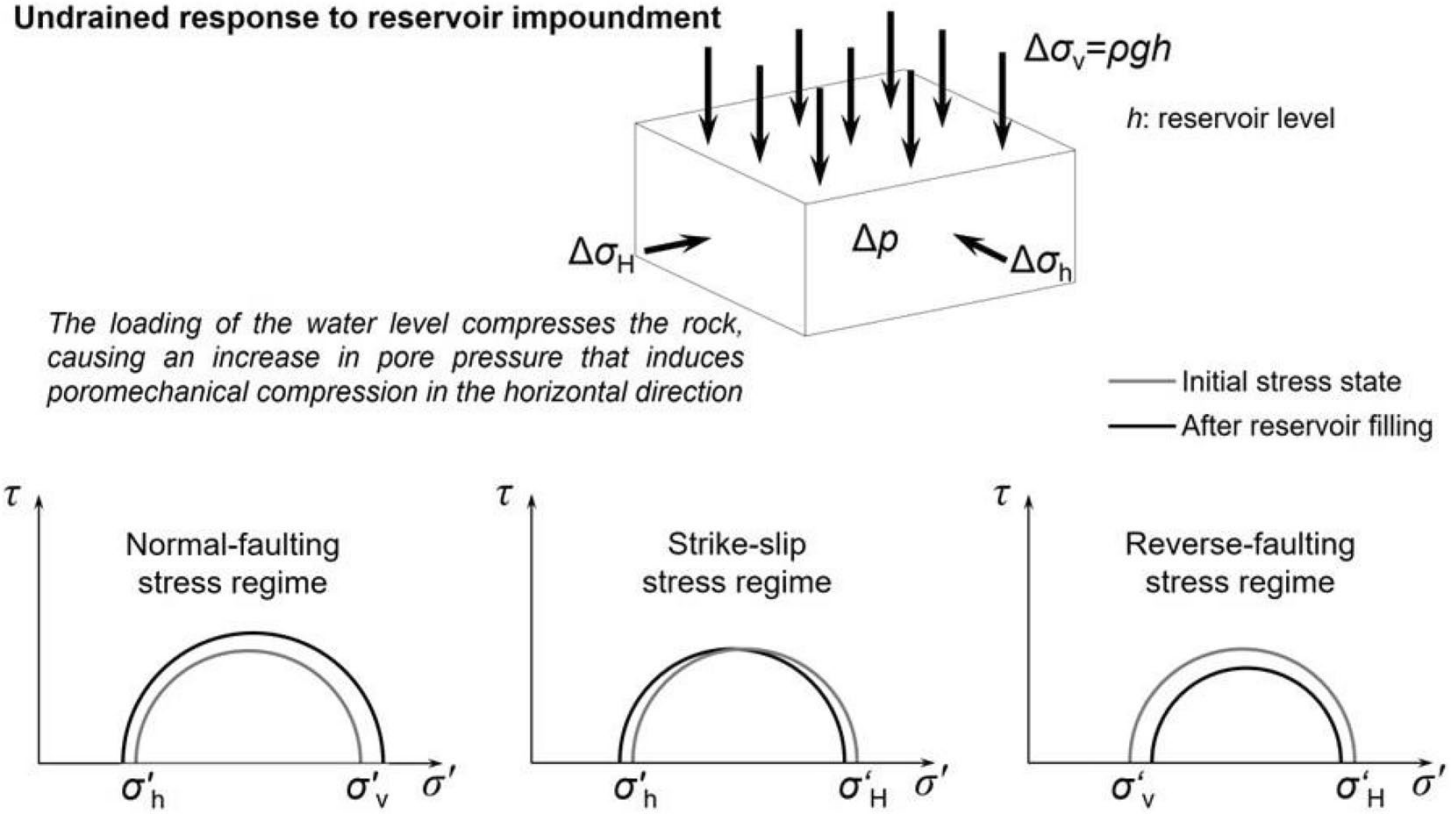
Undrained effective stress changes triggered by reservoir impoundment for normal-faulting, strike-slip, and reverse-faulting stress regimes.

To understand the differences in the ΔCFS between the two nodal planes, pore pressure and stress changes should be analysed. Reservoir impoundment causes a pore pressure increase on the surface that diffuses at a very small rate because of the low permeability of the rock below the reservoir (Fig. 8a). Yet, the pore pressure increase extends further away than the pressure diffusion front. This is a consequence of the undrained response of the low-permeability rock to the reservoir-impoundment loading. As the rock is compressed by the loading of the water column (Fig. 8b), the pore volume is reduced, but the pore water cannot diffuse rapidly and, consequently, pore pressure increases. To satisfy stress equilibrium and displacement compatibility, the horizontal stresses also change in response to the vertical loading and pore pressure changes (Fig. 8c, d). In the schist layer, the horizontal stresses increase in response to the deformation-induced pore pressure build-up. These stress changes induce a stress redistribution around the schist layer that leads to a reduction in the horizontal stresses in the crystalline basement. Such stress redistributions are common in response to stress perturbations induced pore pressure and/or temperature changes^38–40^^.^ Given these stress changes, the deviatoric stress decreases in a reverse-faulting plane because the vertical stress, which is the minimum principal stress, increases more than the maximum horizontal stress (Fig. 9). As a result, fault stability increases, i.e., negative ΔCFS (Fig. 6). In a strike-slip fault, both horizontal stresses have a similar change, so the deviatoric stress is maintained, and since pore pressure increases more than the total horizontal stresses, the effective normal stress decreases (Fig. 9), destabilizing the fault, i.e., positive ΔCFS (Fig. 7).

**Figure 10:**
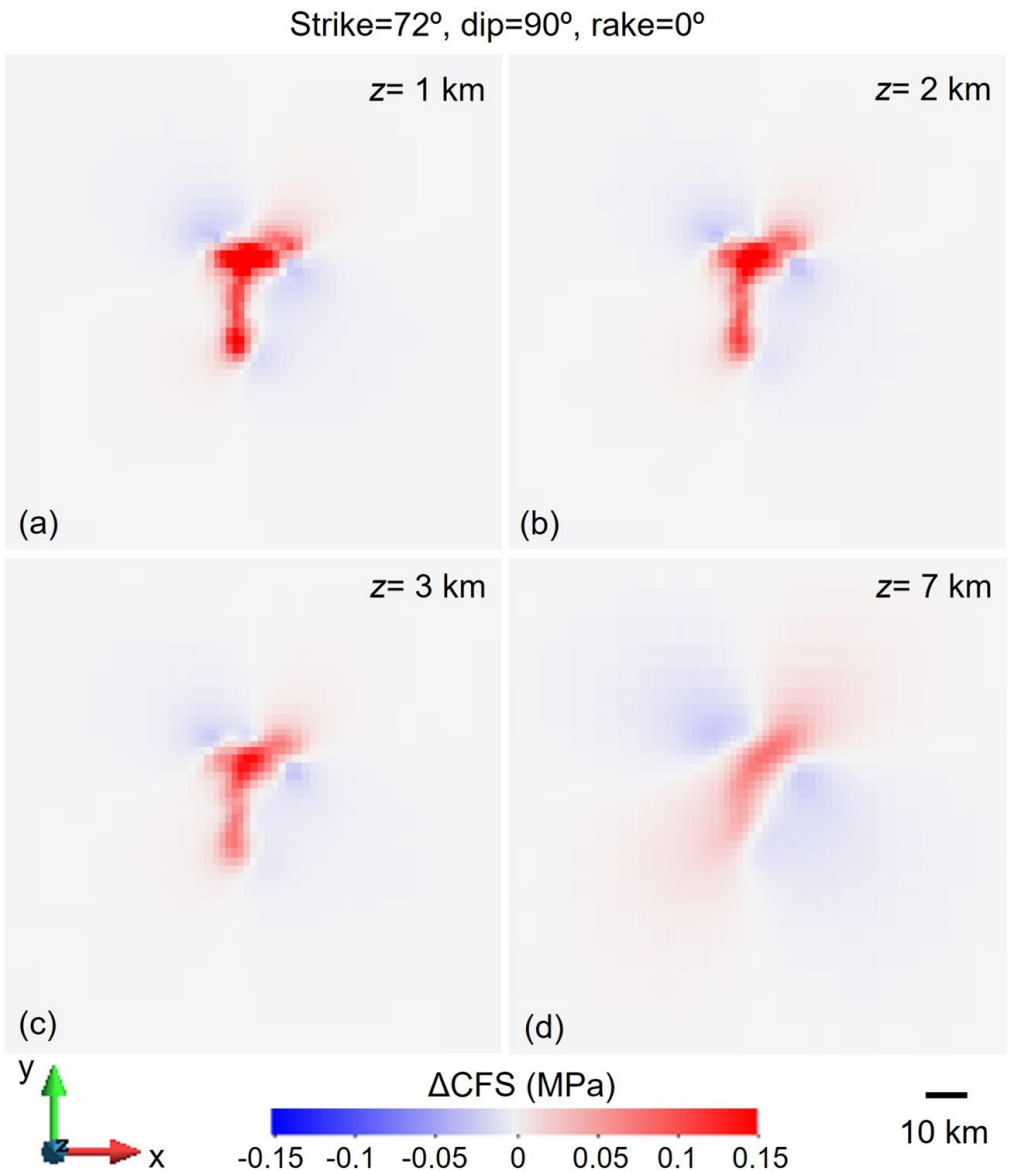
Changes in Coulomb Failure Stress (ΔCFS) for a plane with strike 72°, dip 90° and rake 0°, which is the most critically-stressed plane for the local orientation of the maximum horizontal stress at Nova Ponte, i.e., an azimuth 42°, at the time of the 1998 M4.0 earthquake at (**a**) 1 km, (**b**) 2 km, (**c**) 3 km and (**d**) 7 km deep, for the case of schist permeability equal to 8·10^–15^ m^2^. The ΔCFS has positive values at the location of the earthquake, i.e., southwest of the dam at 3.0 km depth.

There is, to the best of our knowledge, no published focal mechanism of the 1998 M4.0 earthquake. The 1998 M4.0 earthquake was located southwest of the 1995 M3.5 earthquake, with a region with no seismicity between the two earthquakes (Fig. 2). This silent region suggests that the earthquakes were nucleated in different faults or that they belong to different patches of the same fault separated by an aseismic section. In the latter case, the fault orientation could be slightly different from that of the 1995 M3.5 earthquake and needed a higher destabilization, i.e., higher ΔCFS, to nucleate the earthquake. Such higher destabilization can be achieved by (1) re-equilibration of fluids from the compressed to the dilated area^21,41–43^ or (2) a delayed increase in pore pressure by diffusion from the reservoir. We analyse here the second possibility. For a schist permeability of 10^–17^ m^2^, the time required to reach the depth of the 1998 M4.0 earthquake, i.e., 3.0 km deep, is around 3000 years. Thus, a high-permeable pathway, like a permeable fault, should be present to hydraulically connect the reservoir with the fault that nucleated the 1998 M4.0 earthquake. The required permeability to reach 3.0 km in 4.5 years is 6.6·10^–15^ m^2^. Assuming the local orientation of the maximum horizontal stress^30^, i.e., azimuth of 42º, the most critically oriented fault is a vertical strike-slip plane with strike 72º and left-lateral displacement. For such plane, the ΔCFS after 4.5 years of the start of impoundment has positive values below and to the southwest of the reservoir (Fig. 10). Thus, a fault with an orientation similar to this critically stressed plane could have nucleated the 1998 M4.0 earthquake provided that a high-permeability pathway exists between the reservoir and the deep fault.

**Figure 11:**
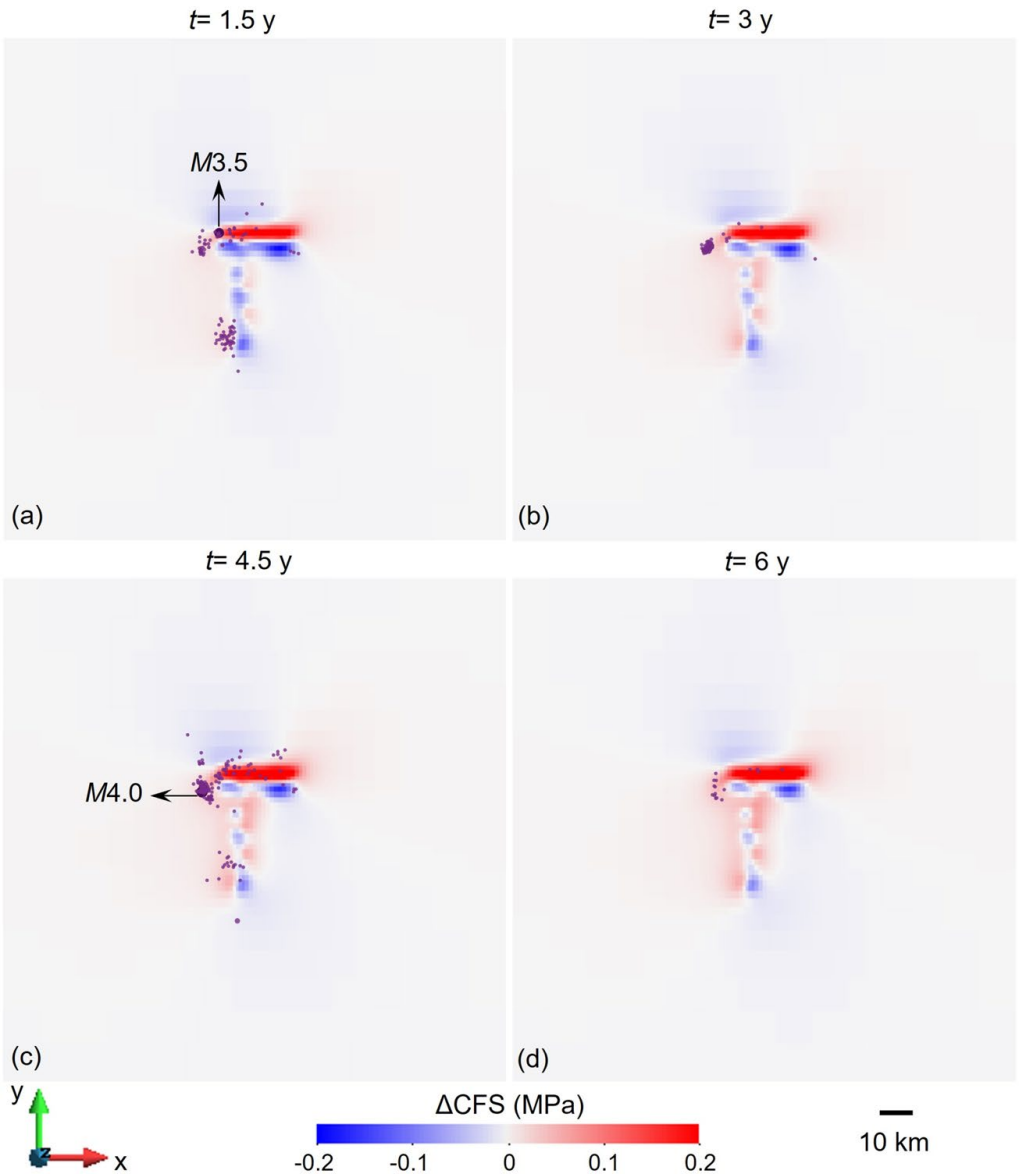
Evolution of the changes in Coulomb Failure Stress (ΔCFS) at (**a**) 1.5, (**b**) 3.0, (**c**) 4.5 and (**d**) 6.0 years after the start of reservoir impoundment, at a depth of 2.5 km for the nodal plane with strike 190°, dip 46° and rake 168° proposed by Marza et al.^31^ for the M3.5 earthquake.

Triggered events are expected to occur within regions undergoing destabilization, i.e., positive ΔCFS values. Given the limited information on the focal mechanism of most events, we compute the ΔCFS in the nodal plane with strike 190º, dip 46º and rake 168º proposed by Marza et al.^31^ for the M3.5 earthquake. Most of the seismicity coincides with the regions with positive ΔCFS (Fig. 11). Such correlation highlights that our model captures the spatiotemporal response of the subsurface to reservoir impoundment at Nova Ponte.

## Discussion

Reducing the risk of induced/triggered seismicity, especially large felt earthquakes, is critical for the successful implementation of dam safety projects, both because felt events have a negative effect on public perception and because large events may pose risks to the dam strength and other infrastructure. A way of managing reservoir-triggered seismicity is to perform site characterization, selection, and continuous monitoring to reduce subsurface uncertainties and carry out a real-time risk assessment of triggered seismicity^43–45^ .

**Figure 12:**
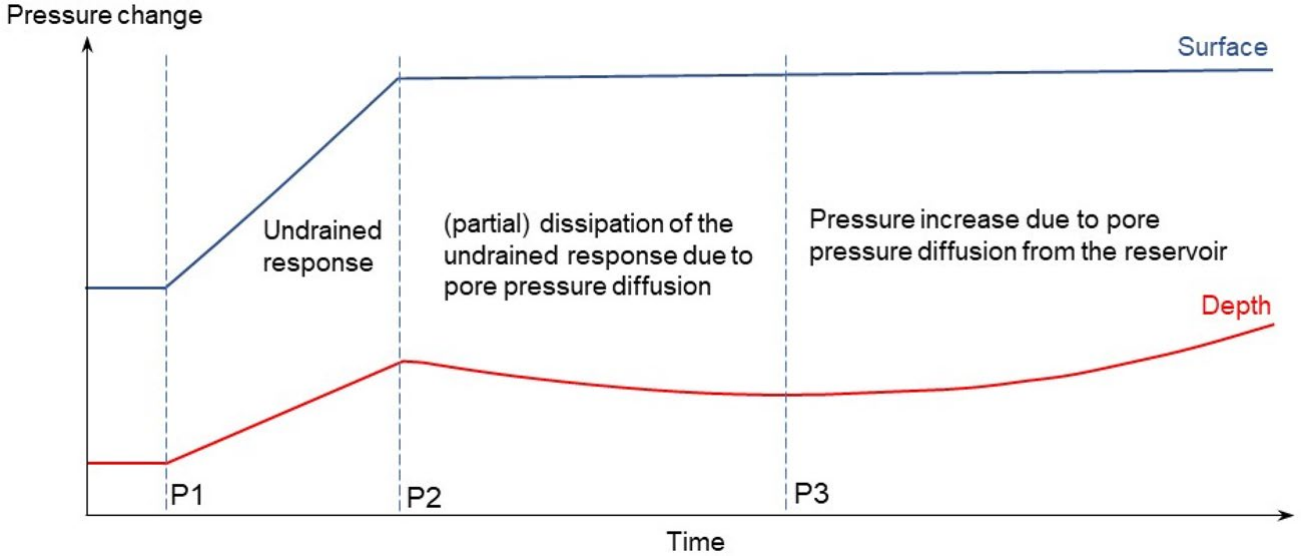
Schematic representation of pore pressure evolution at depth in response to changes in reservoir level: initial pore pressure build up as an undrained response to reservoir impoundment (from P1 to P2), (partial) dissipation of excess pressure (from P2 to P3) and delayed pore pressure increase by pressure diffusion from the reservoir (from P3).

Understanding and forecasting RTS is a challenging task. Two types of stress modifications are believed to be responsible for RTS. One is the rapid undrained response to the loading of the reservoir (from P1 to P2 in Fig. 12) and the other one is the delayed effect due to the pore pressure diffusion (from P3 onwards in Fig. 12)^2^. The delay in the pore pressure build-up due to diffusion depends on the hydraulic diffusivity of the rock. The scheme presented in Fig. 12 can be applied to explain the RTS at the Nova Ponte reservoir. The initial seismicity was likely due to the rock’s undrained response to the reservoir impoundment, while the delayed seismicity that includes the 1998 M4.0 earthquake was due to pore pressure diffusion that brought a pre-existing critically stressed fault to failure conditions. To hydraulically connect the reservoir with the depth of the earthquakes in the Araxá group of schist rock, high-permeability conduits or faults should vertically cross the low-permeability schist. We infer that the vertical permeability of such faults should be at least 6.6·10^–15^ m^2^, which falls within the typical permeability range that explains RTS^5^, i.e., from 5·10^–16^ m^2^ to 5·10^–14^ m^2^. The fact that the nodal plane that better explains the 1995 M3.5 triggered event is a vertical plane (Fig. 7) could explain the presence of such a fault. Nonetheless, faults crossing relatively soft and clay-rich geomaterials usually present a low permeability enhancement along the fault core and damage zone^46^. To better understand the hydrogeological properties of the rock layers below the Nova Ponte reservoir, detailed characterization should be performed to identify the faults that triggered the seismicity and their permeability.

Our study presents an important step forward in the development of predictive seismic hazard models to understand the casual mechanism of the triggered seismicity. By performing numerical simulations of a 3D reservoir model, we compute the pore pressure, stress and stability changes and identify the nodal plane that is more prone to slip at Nova Ponte. In our search for the nodal plane that nucleated the triggered seismicity, we started with a simpler reservoir geometry that consisted of a single rectangle. With such geometry, the fault stability changes induced by the pore pressure and stress changes did not convincingly explain the monitored triggered seismicity. By adopting a T shape of the reservoir (see Figs. 2 and 4), we have found that simulation results show that the nodal plane that triggered the 1995 M3.5 earthquake was the plane with strike 272º, dip 89º and rake 42º (Fig. 7). Our model also reveals the importance of accounting for the different rock types that are found below the reservoir. Figure 8 clearly shows that the stress changes differ significantly at the interface between the Araxá group and the crystalline basement. Including the crystalline basement in predictive models should enable forecasting deep seismicity within the hydraulically disconnected crystalline basement.

The proposed methodology can be used not only to identify and understand the triggering mechanisms of RTS (as we have done in this study), but also to forecast potential triggered earthquakes during site selection, dam design and during the operation of the reservoir. For example, by computing ΔCFS in planes that could be favourably oriented to undergone reactivation given the local stress state (like in Fig. 11), the regions that will be destabilized, i.e., positive ΔCFS, can be identified. Then, efforts on subsurface characterization can concentrate in these regions to identify deep faults that could trigger moderate earthquakes. If faults with potential to trigger damaging earthquakes are identified, the site could be considered of high risk and disregarded during the screening process of finding proper locations for building dams. Otherwise, studies can continue further until the site is considered appropriate. The mapped faults should be included in numerical models to forecast potential RTS because the reactivation of a fault may lead to the destabilization of nearby faults through static stress transfer^43,47^. Thus, including all the available geological and hydrogeological information in numerical models is crucial to increase the accuracy of their predictions^48^. 3D numerical models that solve the coupled hydro-mechanical problem including rock layers and mapped faults can certainly reproduce the two main triggering mechanisms of RTS: the rapid undrained response of the subsurface to loading caused by reservoir impoundment and the delayed pore pressure diffusion. Such models would have been of help in 8 out of the 29 cases of RTS in Brazil which presented initial and delayed RTS^16^. Nova Ponte is one of the classic examples of initial followed by delayed RTS. With a M4.0, Nova Ponte is the second-largest RTS in Brazil, but such magnitude cannot be considered as uncommon and could certainly be exceeded by new dams if a detailed study of the RTS hazard is not performed beforehand.

Managing the risk of RTS requires reliable forecasting models that include the physics of the problem, i.e., capable of reproducing the undrained response of the subsurface to reservoir impoundment and pore-pressure diffusion, and the geological rock layers and features like faults. Building such a model requires a thorough characterization prior to reservoir impoundment, and preferably, before dam construction to identify the potential high hazard of inducing moderate to large earthquakes. Unless the hydro-mechanical properties of the rock layers, i.e., permeability, porosity, and stiffness, are well characterized, as well as the presence of pre-existing faults and their permeability and strength, anticipating undesired RTS is unfeasible. Thus, future efforts should be directed towards gaining comprehensive information of the subsurface to manage RTS.

## Conclusions

We have investigated the causal mechanisms of the RTS at Nova Ponte, which represents the second-largest triggered event in Brazil. Simulation results of a coupled hydro-mechanical 3D model that includes the geological layers of the site have revealed that the nodal plane that nucleated the 1995 M3.5 earthquake was a vertical plane with E–W orientation and reverse left-lateral displacement. This initial seismicity was caused by undrained poromechanical changes of the subsurface in response to reservoir impoundment. In contrast, the delayed seismicity with the 1998 M4.0 earthquake was likely triggered by pore pressure diffusion that pressurized a critically stressed fault at 3.0-km depth. For pore pressure diffusion to reach the depth of the hypocentre, a pathway with permeability higher than 6.6·10^−15^ m^2^ should cross the schist where the earthquake was nucleated. The vertical fault of the 1995 M3.5 event could have enabled pore pressure propagation at depth. Our model highlights the importance of including a realistic geometry to the reservoir as well as a detailed representation of the subsurface, including the rock layers present below the reservoir. Predictive models with such features should be able to reliably forecast RTS.

## Data Availability

The datasets used and/or analysed during the current study are available from the corresponding author upon reasonable request.

## Code availability

The finite element method code used in this study is open source and can be downloaded at https://deca.upc.edu/en/projects/code_bright/downloads.
